# MXene-Based Composites as Nanozymes in Biomedicine: A Perspective

**DOI:** 10.1007/s40820-022-00958-7

**Published:** 2022-11-04

**Authors:** Siavash Iravani, Rajender S. Varma

**Affiliations:** 1 Faculty of Pharmacy and Pharmaceutical Sciences, Isfahan University of Medical Sciences, Isfahan, Iran; 2 Regional Centre of Advanced Technologies and Materials, Department of Physical Chemistry, Faculty of Science, Palacký University in Olomouc, Šlechtitelů 27, 783 71 Olomouc, Czech Republic

**Keywords:** MXenes, MXene-based nanozymes, Therapeutics, Diagnostics, Theranostics

## Abstract

MXene-based nanozymes have garnered considerable attention because of their potential environmental and biomedical applications. These materials encompass alluring and manageable catalytic performances and physicochemical features, which make them suitable as (bio)sensors with high selectivity/sensitivity and efficiency. MXene-based structures with suitable electrical conductivity, biocompatibility, large surface area, optical/magnetic properties, and thermal/mechanical features can be applied in designing innovative nanozymes with area-dependent electrocatalytic performances. Despite the advances made, there is still a long way to deploy MXene-based nanozymes, especially in medical and healthcare applications; limitations pertaining the peroxidase-like activity and sensitivity/selectivity may restrict further practical applications of pristine MXenes. Thus, developing an efficient surface engineering tactic is still required to fabricate multifunctional MXene-based nanozymes with excellent activity. To obtain MXene-based nanozymes with unique physicochemical features and high stability, some crucial steps such as hybridization and modification ought to be performed. Notably, (nano)toxicological and long-term biosafety analyses along with clinical translation studies still need to be comprehensively addressed. Although very limited reports exist pertaining to the biomedical potentials of MXene-based nanozymes, the future explorations should transition toward the extensive research and detailed analyses to realize additional potentials of these structures in biomedicine with a focus on clinical and industrial aspects. In this perspective, therapeutic, diagnostic, and theranostic applications of MXene-based nanozymes are deliberated with a focus on future perspectives toward more successful clinical translational studies. The current state-of-the-art biomedical advances in the use of MXene-based nanozymes, as well as their developmental challenges and future prospects are also highlighted. In view of the fascinating properties of MXene-based nanozymes, these materials can open significant new opportunities in the future of bio- and nanomedicine.

## Highlights

- The development of nanozymes with lower manufacturing cost, higher catalytic stability, and ease of modification than natural enzymes ought to be a priority for scientific research.
- MXene-based nanozymes have attracted considerable attention in the field of bio- and nanomedicine due to their unique catalytic and physicochemical properties.
- Due to the fascinating properties of MXene-based nanozymes, these materials can open up considerable new horizons in the future of bio- and nanomedicine.

## Graphical abstract

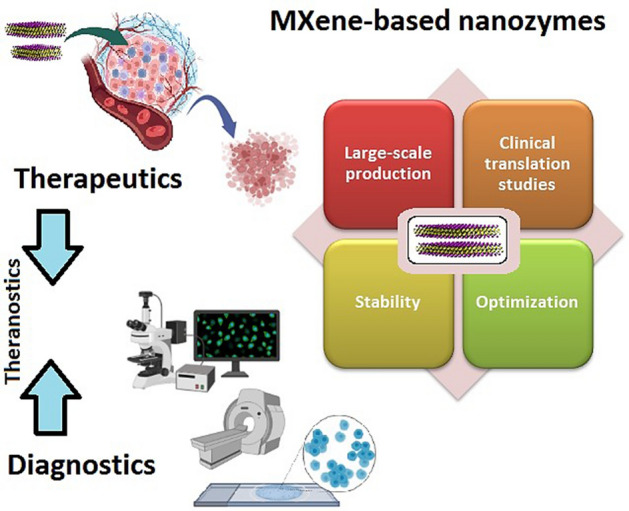

## Introduction

Nanostructured artificial enzymes (nanozymes) have shown promising enzyme-like catalytic features [1], which make them prime candidates for biomedical applications such as biosensing, catalytic therapeutics, cancer theranostics, and immunoassays [2–8]. As an example, to augment the low therapeutic efficacy of ferrotherapy in cancer treatment, a hybrid semiconducting nanozyme with significant efficiency of photothermal conversion was constructed for second near-infrared (NIR) photothermal ferrotherapy guided by photoacoustic imaging [9]. Feng et al. [10] introduced ultrasmall SnFe_2_O_4_ nanozyme for simultaneous photothermal, photodynamic, and chemodynamic cancer therapy. In addition, an injectable nanozyme hydrogel was introduced as reservoir of aggregation-induced emission luminogen as well as release controller for tumor therapy with high efficiency [11]. Among the nanostructures/nanosystems designed for biomedical and catalytic applications, MXenes with unique lamellar structures possess high conductivity properties, and can be applied for improving the photo-electrocatalytic performances of nanocomposites as co-catalysts [4, 12–14]. These materials with excellent photocatalytic activity and photostability have been widely explored in designing a variety of (nano)photocatalysts [15]. In one study, after the formation of magnetic α-Fe_2_O_3_/ZnFe_2_O_4_ heterojunctions through a one-step hydrothermal synthesis, the photocatalyst was prepared utilizing MXenes as co-catalysts through ultrasonic-assisted self-assembly to disperse obtained magnetic heterojunctions on the surface of MXene (Ti_3_C_2_) [16]. Besides, MXene-based structures exhibit a large surface area, high electrical conductivity, excellent functionalization potentials, and electrochemical properties, which make them promising candidates for conductive and energy storage applications [17–20]. They have been broadly explored in the field of bioimaging [21], (nano)sensors [22, 23], battery technology, energy storage [24], electromagnetic interference shielding [12], supercapacitors [25], triboelectric nano-generators, drug delivery [26–29], cancer theranostics [14, 30], desalination, water treatment [31], tissue engineering, regenerative medicine [13], and conductive coatings, among others. This is due to their unique architectures (sheet morphology), excellent potentials in reduction/oxidation reactions, superb metallic conductivity, light weight, optical properties, tunable surface chemistry, unique mechanical features, and easy solution processability [13, 14, 32–34]. Assorted flexible nanozyme sensors have been fabricated for the purpose of intelligent sensing using MXene-based structures [35].

Despite several advantages of natural enzymes such as appropriate catalytic/biological activities and robust substrate specificity, these enzymes suffer from limitations/challenges namely higher costs, poor reusability, low environmental stability, and difficulty in isolation/extraction/purification, thus restricting their large scale biomedical applications [36, 37]. Consequently, studies on enzyme mimics have been investigated to provide a low-cost and highly stable alternative to natural enzymes. Finding nanomaterials with fascinating enzyme-like characteristics comparable to those of catalase, superoxide dismutase, oxidase, peroxidase, etc., have prompted researchers to perform additional studies on functional nanomaterials with biomimetic enzymatic characteristics (termed nanozymes) [38–40]. Compared to natural enzymes, the nanozymes have displayed advantages of cost-effectiveness, longer durability/better reusability, superior chemical stability, robust catalytic activities, and the ease of synthesis/functionalization, which make them promising candidates for biomedical diagnostic, therapeutic, and theranostic applications [36, 41]. With the significant advancements in nano(bio)technology, bio-/nano-catalysis, artificial intelligence science, and computational design, a variety of two-dimensional (2D) material-based functional nanozymes have been introduced based on graphene, transition metal oxide nanosheets, metal–organic frameworks (MOFs), layered transition metal dichalcogenides nanosheets, and MXenes owing to their high surface area, good electronic conductivity, and numerous available active sites [36, 42].

MXene-based composites as nanozymes have been recently explored for environmental applications such as cobalt-doped MXene (Ti_3_C_2_) nanosheets [43] or MXenes/DNA/platinum (Pt) nanocomposites [44], with strong peroxidase-like features as sensing nanosystems with multimodal potentials [20, 35, 45, 46]; however, very limited studies have been explored the biomedical applications of MXene-based composites as nanozymes with advantage of tunable catalytic properties (Table 1). MXenes with unique chemical structures, high surface area, elastic mechanical strength, thermal/electrical conductivity, and optical/mechanical properties have been widely synthesized using chemical vapor deposition [47], hydrothermal fabrication [48], electrochemical production [49], etching techniques, urea glass methods, and bioinspired techniques; the selection of suitable optimization conditions and techniques for the synthesis of MXenes significantly depends on their MAX precursors [19, 50–55]. The construction of distinctly functionalized MXene-based structures with improved adsorption, flexibility, electric/photothermal conductivity, and optical/mechanical properties, offer access to innovative nanozymes with high efficiency and stability deployable for biomedical purposes [56–58]; however, systematic studies ought to be envisioned to uncover challenges and the prospects of this field of science [13, 14, 59–62]. Several studies have reported the enzyme-mimicking activities of MXene-based composites, such as peroxidase (to break down H_2_O_2_), glutathione oxidase (to consume glutathione), and catalase (to produce O_2_ from H_2_O_2_ for enhancement of the photodynamic therapy) [36, 63, 64]. However, intrinsic catalytic activity of MXenes alone (such as their peroxidase-like activities) still needs improvement to be competitive with other nanozymes (such as metals or metal oxides) [7, 43, 65]. Thus, efforts have focused on hybridization of MXenes with other nanomaterials (copper sulfide (CuS), Mn_3_(PO_4_)_2_, or NiFe layered double hydroxide) to improve their catalytic characteristics [66]; noble metal nanomaterials can be employed in designing MXene-metal nanohybrids as enzyme mimetics with enhanced catalytic activities [43, 46, 66, 67]. Herein, biomedical prospects of MXene-based nanozymes with recent advancements, challenges and future directions are deliberated to motivate researchers for additional explorations in this field of science.

**Table 1:** Some selected examples of MXene-based nanozymes with biomedical potential

MXene-based composites	Applications	Advantages/benefits	References
MnO_2_ nanozyme-loaded MXene (Ti_3_C_2_)	Cancer synergistic photothermal-chemodynamic therapy	Enhanced production of reactive oxygen species (ROS) and O_2_	[68]
		Reduction of glutathione overexpression	
		Elimination of tumor cells through the formation of highly toxic ·OH	
Platinum (Pt) decorated MXene (Ti_3_C_2_T_*x*_) nanocomposites	Phototheranostics; hyperthermia-amplified nanozyme catalytic therapy	Generation of ·OH to stimulate the apoptosis and necrosis of cancer cells	[69]
		Suitable photothermal effects along with photoacoustic imaging capabilities	
Pt decorated MXene (Nb_2_C) nanocomposites	Targeted cancer therapy	MXenes improved the nanozyme therapy	[70]
		Excellent tumor regression (in vivo)	
		The catalase- and oxidase-like performances of Pt nanozymes were highly enhanced to generate O_2_ and ROS in combination with tumor-penetrating photothermal therapy	
Dual active nanozyme-loaded Ti_3_C_2_ (MXene)/CeO_2_-polyvinylpyrrolidone nanocomposites	Tumor nanocatalytic therapy combined with photothermal effects	Enhanced tumor growth inhibition (~ 92%)	[71]
		Alleviation of hypoxia and elevation of oxidative stress in the tumor microenvironment	
V_2_C (MXene)-based nanozymes	Ischemic stroke treatment (in vivo)	Multiple enzyme-like performances (mimicking the superoxide dismutase, peroxidase, catalase, and glutathione peroxidase activities)	[72]
		Superoxide anion radical and H_2_O_2_ were catalyzed into water and oxygen, scavenging toxic ·OH to highly suppress the elevation of intracellular ROS	
		MXenes as nanoscale contrast agents for T_1_-weighted magnetic resonance imaging (MRI)	
Cobalt ferrite/MXene nanozymes	Drug-free synergistic phototherapy and multi-enzyme-mimicking catalytic nanotherapy against bacterial infections (in vitro and in vivo)	Excellent biocompatibility (in vitro and in vivo)	[63]
		Enhanced synergistic photothermal/photodynamic therapy, mimicking peroxidase, glutathione oxidase, and catalase activities	
		Targeted phototherapy to raise the bacterial membrane permeability and enhance the ROS level in bacterial cells	

## Biomedical Prospects

### Therapeutics

**Fig. 1:**
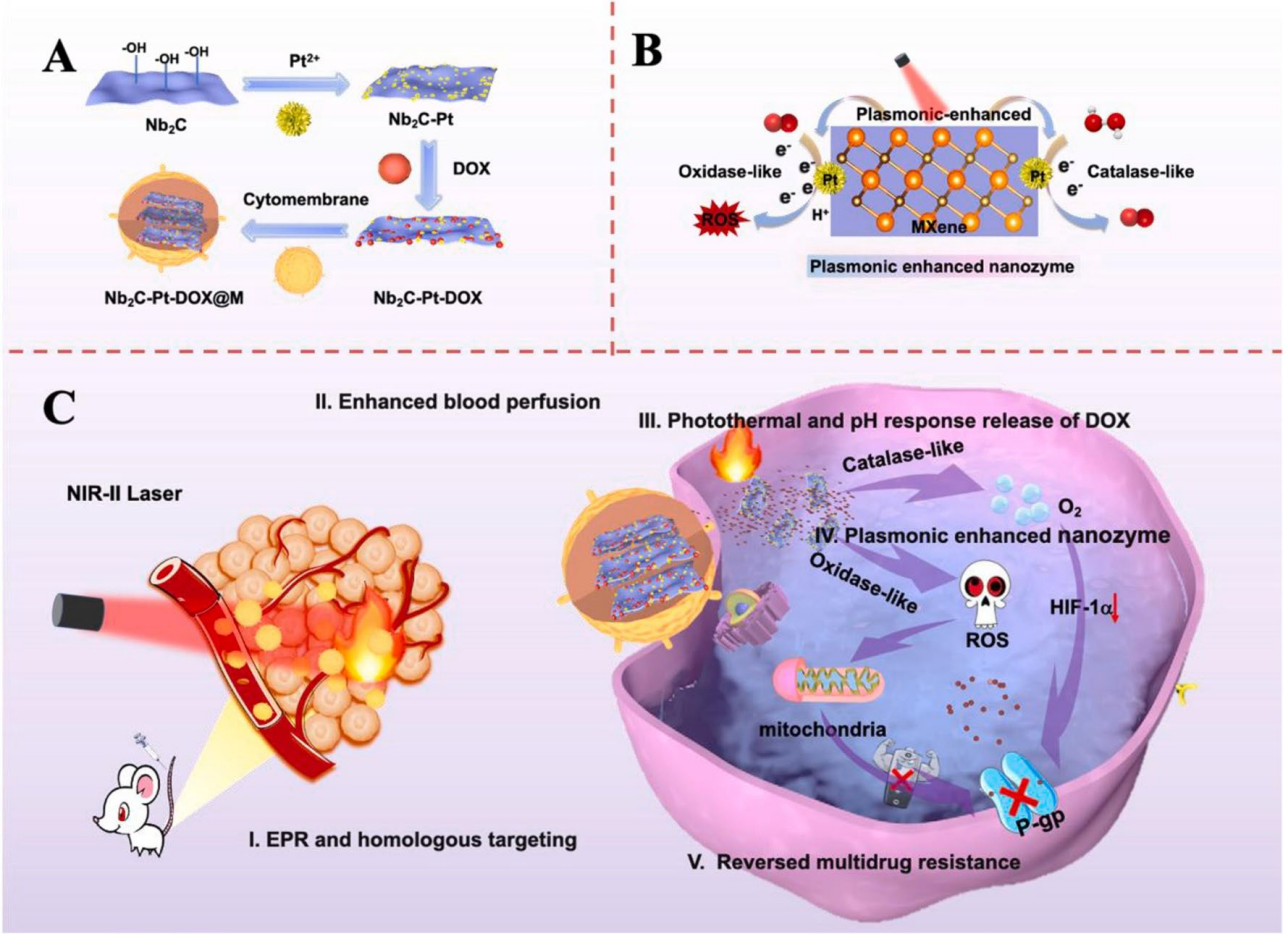
**A** The preparative process for biomimetic photo-induced plasmonic assembly for targeted cancer nanotherapy in NIR-II bio-window (in vivo). **B** The catalase- and oxidase-like performance of Pt nanozyme, and **C** related mechanism of drug release and tumor suppression (**I**–**V**). *M* MXene, *DOX* doxorubicin. Reproduced with permission from Ref. [70]. Copyright 2021 Elsevier

MXenes have been applied for development of nanozyme-based catalysts, offering attractive capabilities in the field of biotherapy and immunoassay. Notably, MXenes with inherent photothermal activities and suitable photostability (under laser irradiation) revealed plasmon-enhanced photocatalytic features, which render them alluring candidates for effective photo-responsive nanomedicine [15]. For instance, a novel strategy was introduced based on plasmonic enhanced nanozymes via the construction of biomimetic photo-induced plasmonic assembly consisting of MXenes (Nb_2_C), Pt nanozyme, anticancer drug (doxorubicin), and tumor cytomembrane (Fig. 1) [70]. Accordingly, after homologous targeting and internalization into tumor cells, the hot-electrons could be excited from MXenes under NIR-II laser irradiation, facilitating the catalase- and oxidase-like performances of Pt nanozyme to form O_2_ and reactive oxygen species (ROS) in concert with tumor-penetrating photothermal nanotherapy. In addition, under hyperpyrexia and acidic conditions, the release of doxorubicin was enhanced by inhibiting P-glycoprotein-mediated drug efflux ensued by ROS and O_2_. Compared to the pristine nanozyme, this MXene-based nanozyme could efficiently reduce the viability of HeLa cells (~ 38.67%), offering a novel nanozyme-based treatment strategy with improved tumor suppression. Such biocatalysis-based nanotherapy tactics deploying MXene-based biomimetic plasmonic assembly should be further evaluated, especially for targeted cancer nanotherapy [70].

**Fig. 2:**
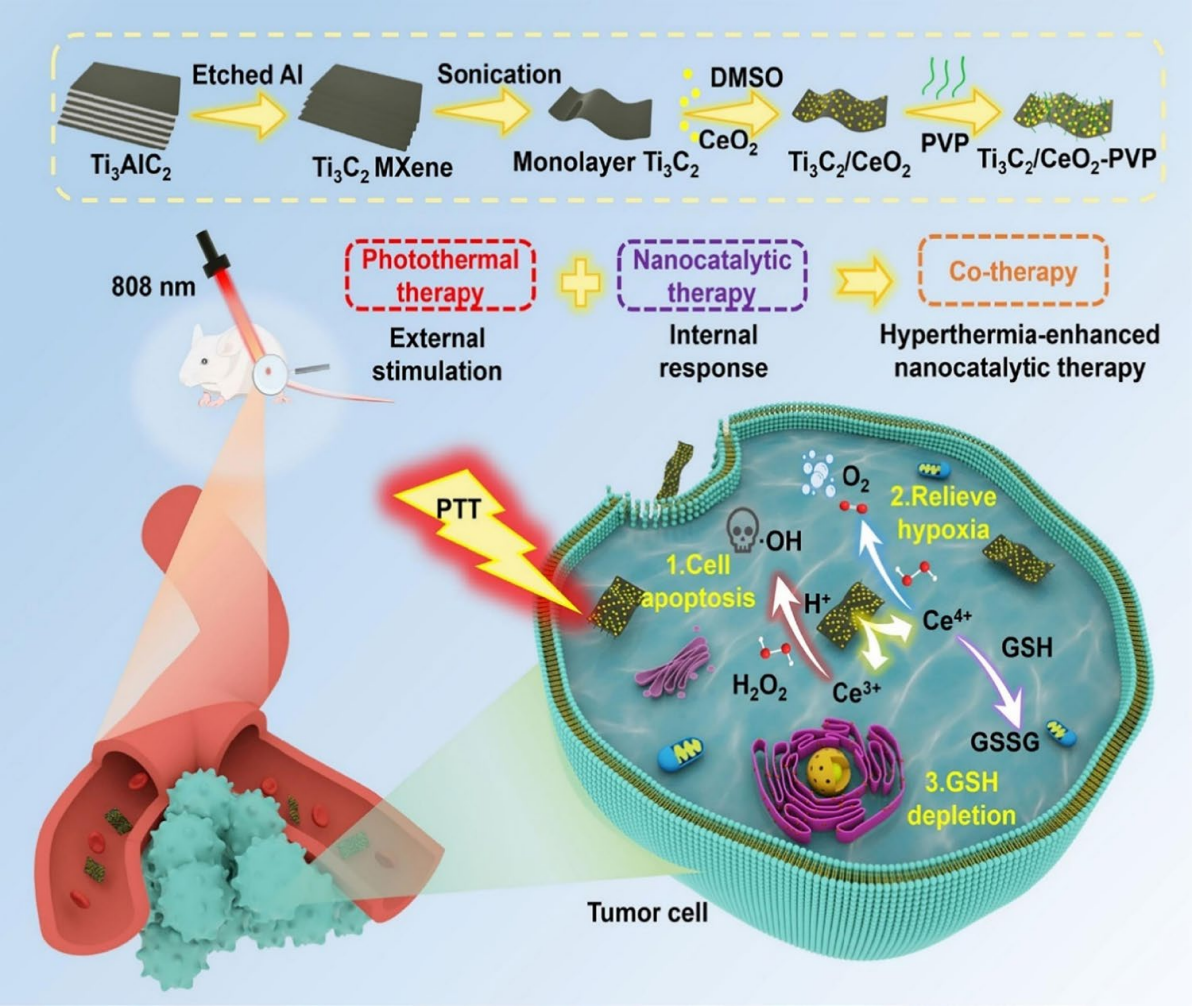
MXene (Ti_3_C_2_)/CeO_2_-polyvinylpyrrolidone nanocomposites with photothermal effects, strong catalytic activities, and glutathione degradation capabilities exhibited suitable applicability for hyperthermia-enhanced tumor combinational therapy (in vivo). *DMSO* dimethyl sulfoxide, *PTT* photothermal therapy, *GSH* glutathione, *PVP* polyvinylpyrrolidone. Reproduced with permission from Ref. [71]. Copyright 2022 Elsevier

To overcome the low activities of nanozymes in the tumor microenvironment that may cause the restricted therapeutic effects, MXene (Ti_3_C_2_)/CeO_2_-polyvinylpyrrolidone nanocomposites with photo-enhanced dual enzyme performances (promoting catalase and peroxidase) were constructed for synergistic tumor therapy (Fig. 2) [71]. The catalase- and peroxidase-like performance of these MXene-based nanozymes alleviated hypoxia and elevated oxidative stress in the tumor microenvironment; they also exhibited excellent capability for the degradation of glutathione to improve the tumor ablation. These nanozymes could generate large amounts of ·OH via the catalytic decomposition of hydrogen peroxide (H_2_O_2_) in the tumor microenvironment, causing apoptosis of tumor cells. The photothermal effects and dual enzyme-like functions could result in improved tumor nanotherapy (the inhibitory effect of tumor growth was ~ 92%), paving the way for efficient nanozyme catalytic therapy [71]. Similarly, photothermal ablation of tumors by warming along with the increased ROS, O_2_ formation, and glutathione reduction could alleviate the hypoxia of tumors and promote catalytic treatments with MnO_2_ nanozyme-loaded MXenes. These nanosystems can be harnessed for bimodal photothermal-chemodynamic cancer therapy with good biocompatibility and high efficiency of tumor ablation [68].

**Fig. 3:**
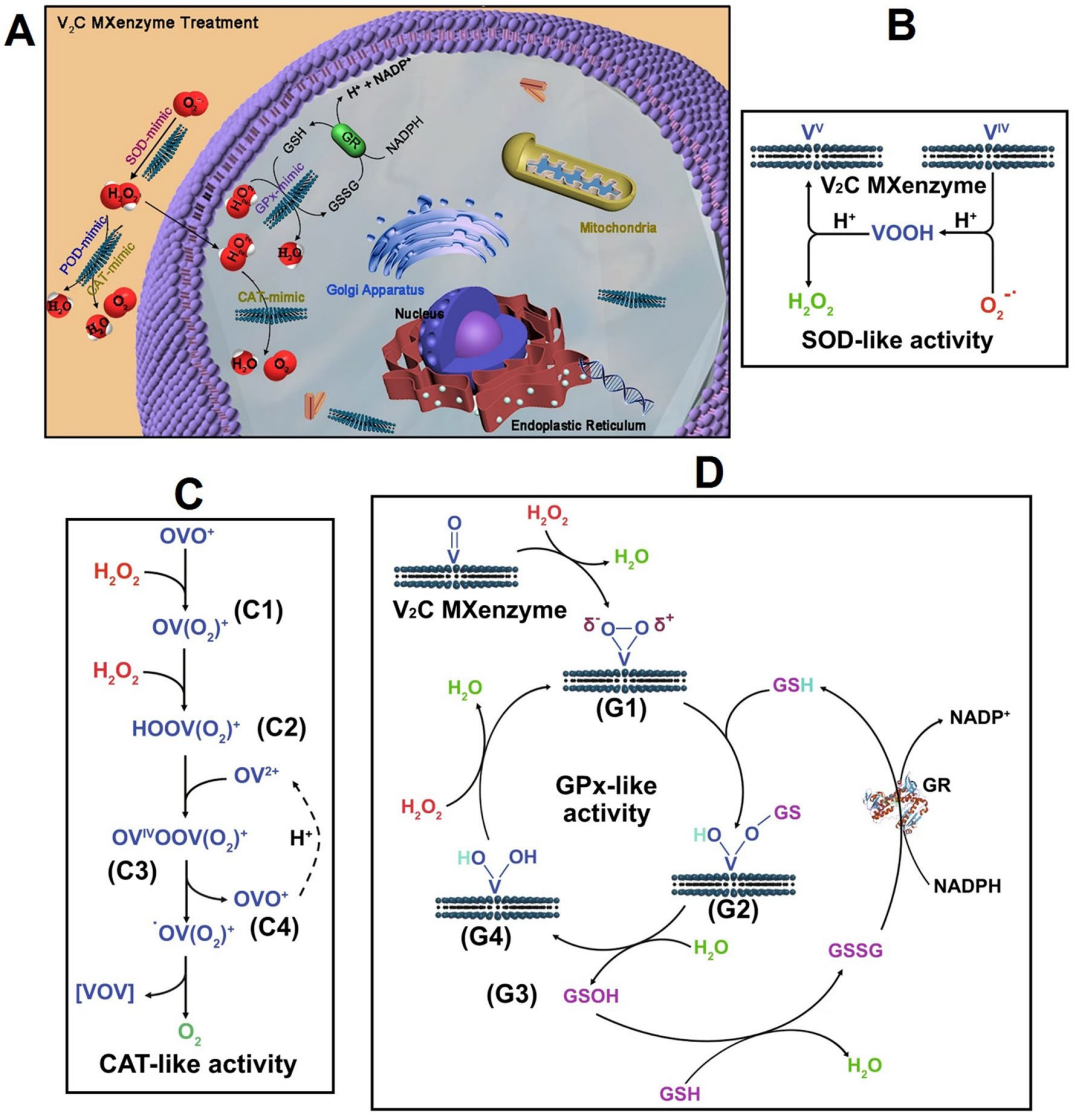
**A** V_2_C MXenzymes for the treatment of ROS-mediated damages, which could effectively catalyze V2C O_2_.^−·^ into O_2_ and H_2_O_2_, decompose H_2_O_2_ into O_2_ and H_2_O, and eliminate ·OH. **B** The related mechanism of superoxide dismutase (SOD)-like performance of the MXenzyme. **C** The associated mechanism of catalase (CAT)-like performance of the MXenzyme. **D** The related mechanism of glutathione peroxidase (GPx)-like performance of the MXenzyme. *POD* peroxidase, *NADP* nicotinamide adenine dinucleotide phosphate, *GR* glutathione reductase, *GSSG* oxidized glutathione, *GSH* reduced glutathione. Reproduced with permission from Ref. [73]. Copyright 2021 Springer Nature (CC BY 4.0)

MXene-based nanozymes (named MXenzyme) constructed from 2D vanadium carbide (V_2_C) MXene could serve as remarkable multifunctional inorganic analogs of thiol and glutathione peroxidase, catalase, haloperoxidase, peroxidase, and superoxide dismutase, mimicking naturally occurring enzymes along with the intracellular antioxidant defense system against serious oxidative damages mediated by ROS such as lipid peroxidation, DNA damages, and protein carbonylation. Based on the fascinating enzyme-mimicking characteristics of the MXenes, they have been contemplated as attractive candidates for neoteric catalytic biomedicine [73] as they exhibited high biocompatibility (both in vitro and in vivo) with efficient cytoprotection against oxidative stress (in vitro), introducing MXenzyme for the redox homeostasis without disturbing the endogenous antioxidant status. However, future explorations ought to focus on relieving the damages mediated by ROS to pave a way for in vivo treatment of neurodegenerative diseases as well as ROS-mediated damages/inflammatory (Fig. 3) [73]. Since the enzyme-mediated enhancement of ROS at the tumor sites is one of the efficient techniques for modulating intracellular redox status to treat cancers, a camouflaged bionic cascaded-enzyme nano-reactor was designed deploying nanosheets of MXene (Ti_3_C_2_) for combinational tumor phototherapy/enzyme dynamic therapy along with the deoxygenation-activated chemotherapy (hypoxia-activated chemotherapy) [74]. The chemical conjugation of chloroperoxidase and glucose oxidase was performed onto MXene nanosheets loaded with tirapazamine (an anticancer drug). The designed MXene-based nanocomposites could embed into nano-sized cancer cell-originated membrane vesicles with high-expressed CD47 (meTGCT). After the internalization of nanosystems into tumor cells, the cascade reaction of glucose oxidase and chloroperoxidase generated hypochlorous acid (HClO) for enzyme dynamic therapy with high efficiency. Additionally, laser irradiation accelerated the rate of catalytic reactions and increased the formation of singlet oxygen (^1^O_2_). Notably, local hypoxia environment with the oxygen depletion by enzyme dynamic therapy activated the deoxygenation-sensitive prodrug for chemotherapy [74].

### Diagnostics

**Fig. 4:**
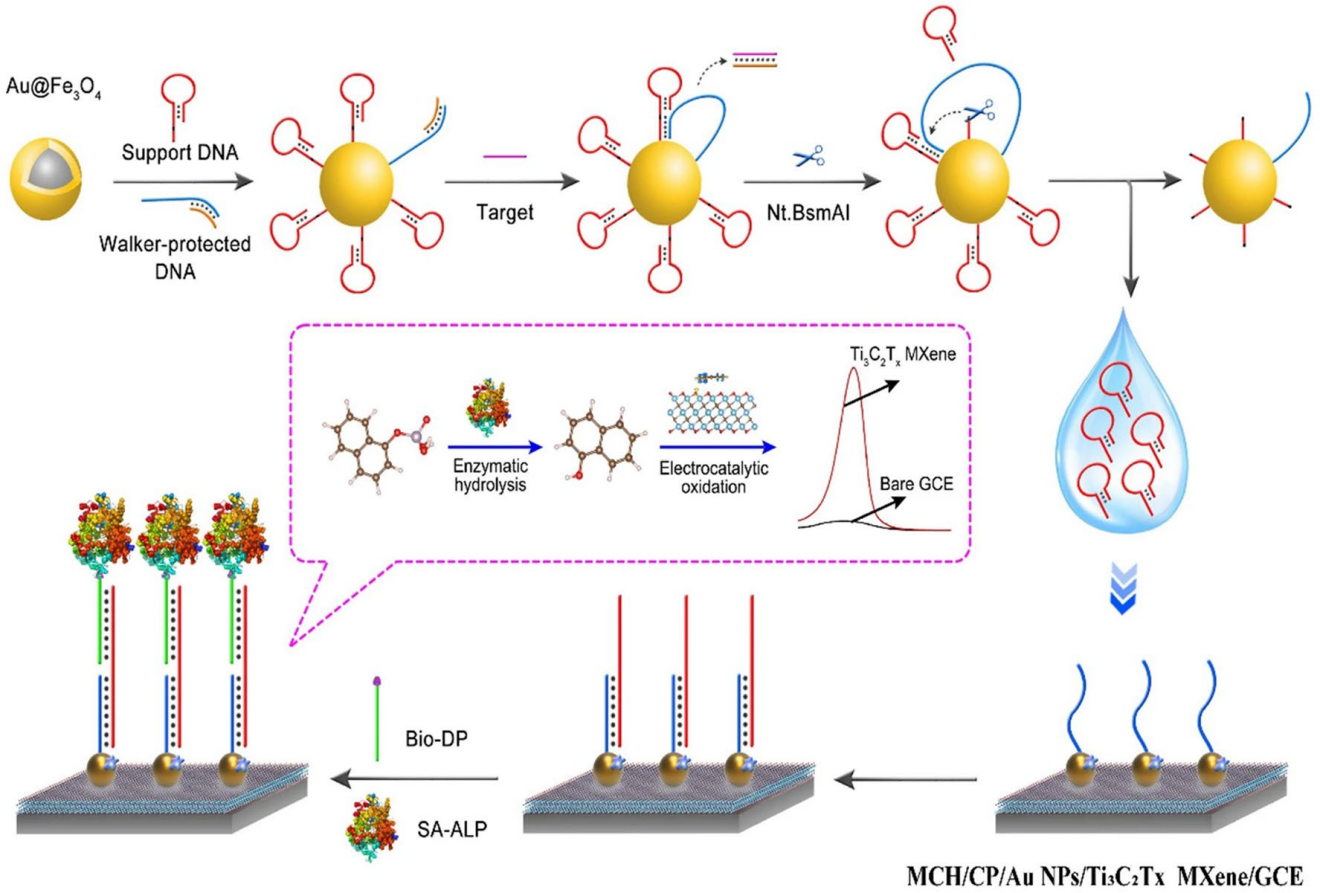
The working principles of an electrochemical biosensor constructed from 6-mercaptohexanol (MCH), Au NPs, MXene (Ti_3_C_2_T_*x*_), and glassy carbon electrode (GCE). Nt.BsmAI nicking endonuclease (Nt.BsmAI). Reproduced with permission from Ref. [65]. Copyright 2022 Springer Nature (CC BY)

The combination of MXenes (Ti_3_C_2_T_*x*_) with alkaline phosphatase could provide cascading catalytic amplification technique utilizing 1-naphthyl phosphate as a substrate, thus resulting in electrochemical signal amplification with high efficiency (Fig. 4) [65]. Accordingly, on the 2D plane, MXenes (Ti_3_C_2_T_*x*_) displayed a suitable area-dependent phenol adsorption with high efficiency to catalyze the electrochemical oxidation; they could be applied for oxidation of phenolic compounds. Also, on an electrode with biosensing application, the MXene was distributed and further decorated with gold (Au) nanoparticles (NPs) to immobilize the DNA capture probe. The designed electrochemical biosensor based on this technique was further exploited for detecting BCR/ABL fusion gene, resulting in superb sensitivity (~ 0.2 fM-20 nM) and limit of detection (LOD) down to ~ 0.05 fM. The biosensor exhibited excellent potential for specifically detection of fusion gene for the initial recognition of acute lymphocytic leukemia and chronic myelogenous [65]. Besides, enzyme-free electrochemical immunosensor was fabricated utilizing palladium (Pd), Pt, nonmetallic elements (boron and phosphorus), MXenes, and CuCl_2_ nanowires for specific detection of kidney injury molecule-1 in the urine [75]. These MXene-based nanocomposites with large surface area and excellent peroxidase-like catalytic performance exhibited suitable analytical activity in the presence of H_2_O_2_. Notably, CuCl_2_ nanowires were combined with biocompatible Au NPs to alter the glassy carbon electrode, and a sandwich-type electrochemical immunosensor was prepared with outstanding electrochemical performances with a good linear response (0.5–100 ng mL^−1^) and LOD of 86 pg mL^−1^, thus providing biosensor with high specificity/selectivity for clinical diagnostics [75].

MXene-based nanocomposite catalysts were designed for intracellular biosensing purposes [76]. In one study, MXene-based nanocomposites were assembled using Au, Pt, and Ti_3_C_2_Cl_2_, providing peroxidase and oxidase mimic activities. They were deployed as colorimetric platforms for in situ sensing of H_2_O_2_ released from live HeLa cells (the detection range = 50–10,000 μM, LOD = 10.24 μM) and colorimetric recognition of glutathione (the detection range = 0.1–20 μM, LOD = 0.07 μM) [76]. In addition, a nanosystem based on MXene (Ti_3_C_2_T_*x*_)-derived TiO_2_/carbon quantum dots was prepared through a hydrothermal treatment of tiny and few-layered MXene nanosheets for specific nanozyme-based colorimetry [77]. The oxygen vacancy in TiO_2_ on the surface of the carbon matrix facilitated the adsorption of O_2_ in the solution and generated ROS to rapidly oxidize 3,3′,5,5′-tetramethylbenzidine without the presence of H_2_O_2_. After inserting glutathione, the oxidized form of 3,3′,5,5′-tetramethylbenzidine was capable of being restored to 3,3′,5,5′-tetramethylbenzidine, causing a reduction in the UV/Vis absorbance value (at 652 nm). This nanozyme-based assay exhibited improved specificity and excellent sensitivity with a LOD of ~ 0.2 μM, thereby opening new window for the specific detection of glutathione in biological mediums (like human serum) [77].

**Fig. 5:**
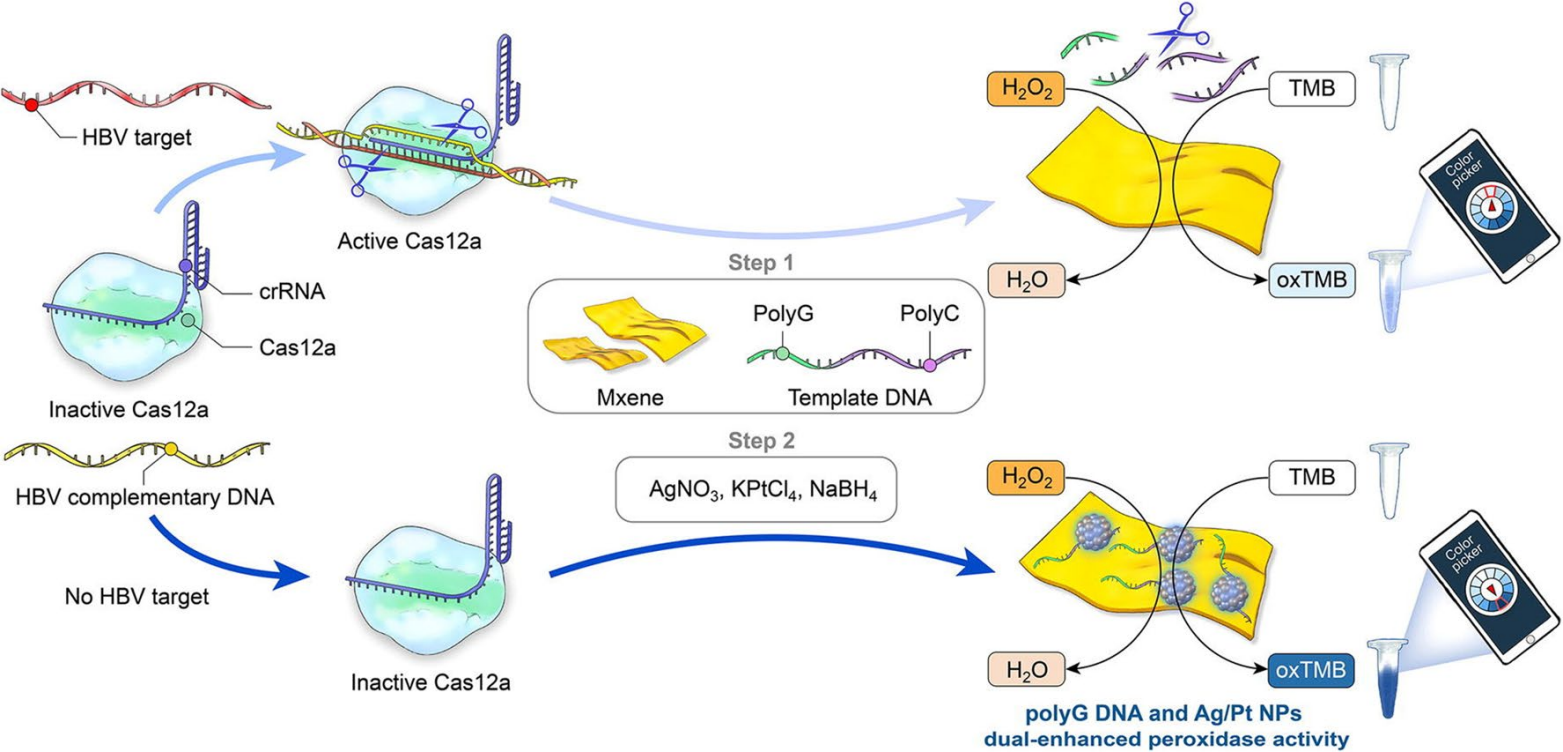
The principles of CRISPR-Cas12a based colorimetric biosensor designed for specific detection of hepatitis B virus (HBV) DNA using MXene-probe DNA-Ag/Pt nanohybrids with catalytic performance. TMB: 3,3ʹ,5,5ʹ-Tetramethylbenzidine. Reproduced with permission from Ref. [67]. Copyright 2022 Elsevier

For label-free and colorimetric sensing of proteins, MXenes have been applied exploiting the unique properties such as their tunable versatile features. Notably, the intrinsic peroxidase-like performance of MXene nanosheets could be improved via the adsorption of single-stranded DNA (ssDNA) on their surfaces [78]. A simple label-free sensing tactic was designed for specific colorimetric recognition of biomolecules (thrombin as a model) using MXene nanosheets (as peroxidase mimic nanozymes) and ssDNA aptamers (as enhancement factors for enzymatic performance) [78]. The ssDNA aptamers were desorbed from MXene nanosheets in the presence of target biomolecules, because of the precise target-aptamer bindings, thereby decreasing the catalytic performance. The designed biosensor (a linear range = 1.0 × 10^−11^–1.0 × 10^−8^ M) demonstrated satisfactory results after testing for real blood samples, signifying that MXenes can be considered as promising nanozymes for targeted detection of biomolecules [78]. In addition, colorimetric biosensor based on CRISPR-Cas12a was introduced for specific detection of hepatitis B virus by applying probe DNA regulation of the catalytic performance of MXene-probe DNA-silver (Ag)/Pt nanohybrids (Fig. 5) [67]. The Cas12a trans-cleavage performance could be successfully activated to degrade the DNA probes in the presence of hepatitis B virus target, thereby inhibiting DNA metallization and enzyme activity enhancer DNA adsorbed on MXene to obtain highly decreased catalytic performances. This colorimetric sensing strategy with high sensitivity/specificity, good accuracy, and stability could be combined with the smartphone platform, permitting visible recognition of target DNA with high sensitivity [67].

Nitrogen and sulfur co-doped MXene (Ti_3_C_2_) nanosheets with excellent peroxidase-like activity and electrochemical activity were deployed to construct a combined colorimetric and electrochemical sensing platform for sensitive detection of uric acid [79]. It was revealed that nitrogen and sulfur doping provided additional active sites and improved the efficiency of electron transport, offering platform with great analytical performance. The uric acid was specifically detected in the range of 2–400 μM with LOD of ~ 0.19 μM [79]. Besides, the quenching performance of MXenes was illustrated by their combinatory utilization with single atomic site cobalt (Co) catalysts in UiO-66 metal–organic frameworks for developing an immunoassay technique for cardiac troponin I on an immunochromatographic test strip platform [80]. These Co single atomic site catalysts exhibited significant enhancement effect on luminol chemiluminescent emission. As a result, the dynamic range for quantification of cardiac troponin I was ~ 1.0–100 pg mL^−1^, with LOD of ~ 0.33 pg mL^−1^ [80].

### Theranostics

**Fig. 6:**
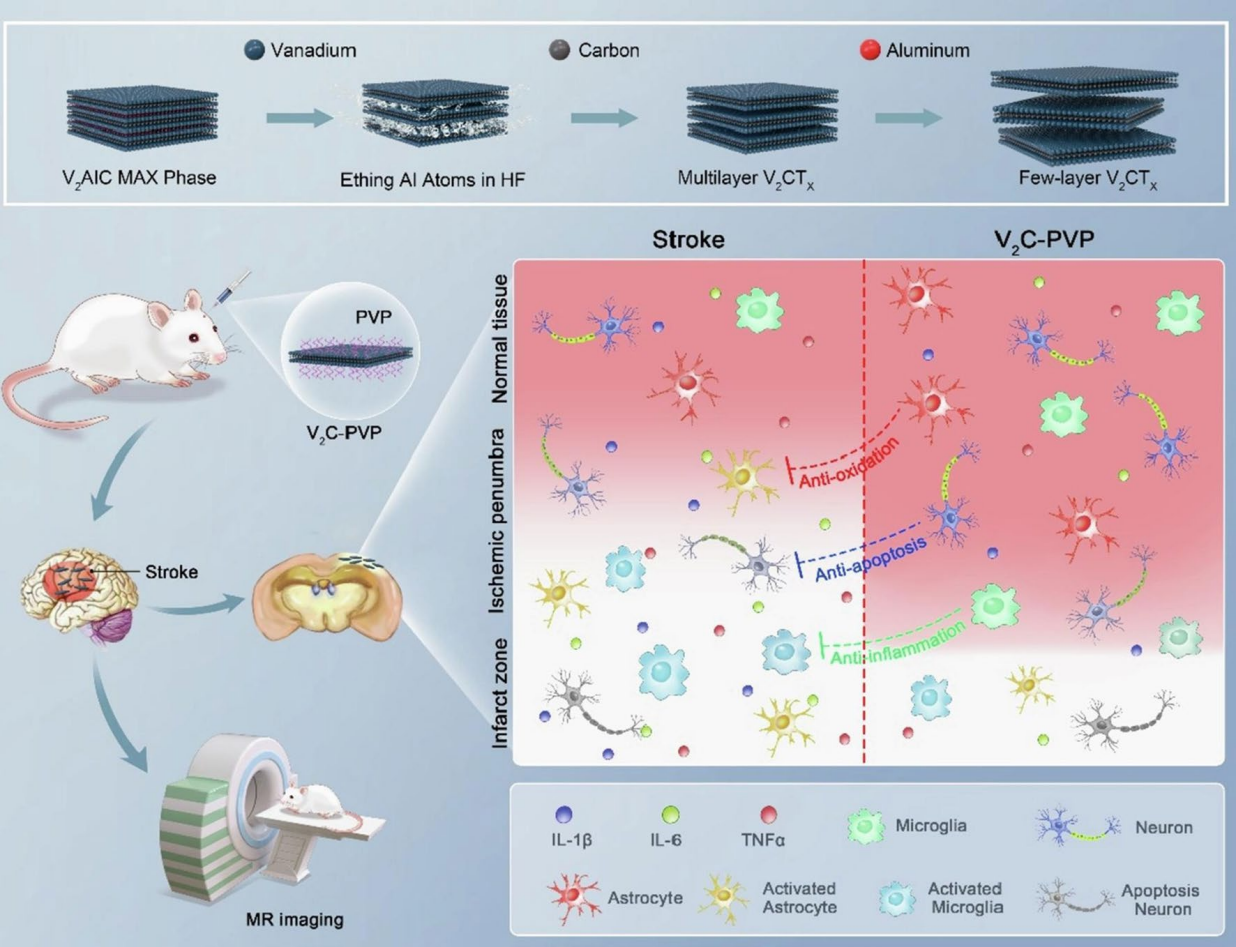
MXene (V_2_C)-based nanozymes with intrinsic multiple enzyme-like performances as theranostic nanoplatforms for treating ischemic stroke through the alleviation of oxidative stress, suppression of cell apoptosis and reduction of inflammation. *HF* hydrogen fluoride, *PVP* polyvinylpyrrolidone; *MR* magnetic resonance. Reproduced with permission from Ref. [72]. Copyright 2022 Elsevier

MXene (V_2_C)-based nanozymes were constructed with theranostic potential for treating ischemic stroke; these nanozymes exhibited excellent capabilities to exert neuroprotection effects by scavenging ROS toward ischemic stroke (Fig. 6) [72]. The MXenes fabricated via etching and delamination processes demonstrated the inherent multiple enzyme-mimicking features and excellent antioxidative capabilities to catalyze toxic/harmful ${\text{O}}_{2}^{\cdot - }$ into nontoxic water and oxygen molecules and scavenge highly toxic ·OH, significantly overwhelming the elevation of ROS. These MXene-based nanozymes protected the central nervous system against ischemic stroke injury through the anti-inflammatory, antiapoptotic, antioxidative effects with no noticeable toxicity or adverse effects. On the other hand, they could function as contrast agents for in vitro/in vivo magnetic resonance imaging (MRI), offering MXene-based theranostic nanozymes with excellent therapeutic efficacy toward ROS-related brain diseases or other ROS-related inflammatory diseases [72]. Zhu et al*.* [69] decorated Pt artificial nanozymes on the MXene (Ti_3_C_2_) nanosheets to obtain nanocomposites for phototheranostic applications. Pt NPs exhibited peroxidase-like activities in the tumor microenvironment to catalyze (in situ) H_2_O_2_ for generating hydroxyl radicals (^·^OH) to stimulate cell apoptosis and necrosis. Notably, these nanocomposites illustrated suitable photothermal effects upon NIR-II light irradiation with a low power density (0.75 W cm^–2^). The peroxidase-like activity was highly improved by the increased temperature ascending from the photothermal effects of Ti_3_C_2_T_*x*_, offering synergistic photothermal/enzyme therapy with photoacoustic imaging benefits [69].

## Biosafety Aspects

Despite the fascinating applications of MXenes and their derivatives, their toxicity and potential environmental risks ought to be systematically analyzed [81, 82]. Although several biocompatible MXene-based composites have been introduced, more explorations are still necessitated for the comprehensive in vitro/in vivo evaluations of their toxicity and biosafety issues [81]. In this context, toxicological, cytotoxicity, and biocompatibility properties are crucial aspects for clinical translation of MXene-based nanozymes in biomedicine [83–87]. Overall, physicochemical features of these materials along with their cellular interaction and accumulation in targeted sites can significantly affect their possible toxic effects [88]. Thus, methodical toxicological and cytotoxicity assessments (both in vitro and in vivo) as well as clinical translation studies are highly demanded, especially regarding their endocytosis, ROS/oxidative stress, penetration/attachment, DNA damages, apoptosis, inflammatory reactions, etc. [59, 62, 89, 90]. It was revealed that MXenes caused toxicity on zebrafish embryo models (in vivo) with dose dependent behavior [81]; however, no noticeable teratogenic effects could be detected at 100 μg mL^−1^. On the other hand, neurotoxicity evaluations revealed that MXenes had no meaningful toxic effects on neuromuscular activities at 50 μg mL^−1^. They are categorized as practically non-toxic structures at concentrations below 100 μg mL^−1^, based on the Acute Toxicity Rating Scale by the Fish and Wildlife Service [81].

Possible toxic effects of MXenes have been evaluated on the early stage of the embryo [91]. MXenes could adversely affect the early stage of embryogenesis, since ~ 46% of MXene-exposed embryos died during 1–5 days after exposure. They inhibited angiogenesis of the chorioallantoic membrane of embryo after 5 days incubation, showing possible toxicity of these structures on the early stage of embryogenesis [91]; however, still more explorations are necessary to address the related toxicity mechanisms along with the other crucial aspects regarding their long‐term biosafety, biodegradation, biocompatibility, dispersibility, and solubility [91]. In one study, after hemocompatibility and excretion analysis of MXene (Ti_3_C_2_)-soybean phospholipid structures, no noticeable acute toxicity and high histocompatibility could be detected; these materials are normally excreted out of the body through feces and urine with total excretory amount of ~ 10.35% [89]. Besides, after biocompatibility assessment of surface-functionalized MXenes, no noticeable defects could be identified in hematological indexes, behavior, biochemical factors, and body weight of examined mice, showing no chronic pathological toxic effects [62]. MnO_*x*_/MXene (Ti_3_C_2_) composites functionalized with soybean phospholipid displayed improved stability along with high biocompatibility and dispersibility, thus introducing suitable candidates for clinical purposes [92].

## Challenges and Perspectives

In addition to toxicity and biosafety issues, surface modification/functionalization, environmentally benign synthesis techniques, optimization conditions, and large scale production are important challenging issues that need to be further explored [84, 93, 94]. Several crucial parameters such as the concentration and chemical structures can significantly affect the optical, mechanical, electronic, magnetic, and thermal properties of MXenes and their composites [95]. Designing simple, cost-effective, and eco-friendly synthesis techniques with high yield and low-cost benefits ought to be further explored, especially to find real-life applicability and commercial viability of MXenes and their derivatives in clinical and biomedical applications [83, 96, 97]. Challenging issues regarding the stability of MXenes and their possible oxidation or aggregation ought to be taken into account. Surface functionalization using suitable functional groups would also help to improve the stability of MXenes [98].

Suitable hybridization using polymers, carbon materials, and other inorganic materials can significantly improve the stability and functionality of MXene-based composites [99–101]. To reduce or prevent the oxidative decomposition, crucial parameters regarding the synthesis and storage conditions (e.g., pH, storage media, temperature, and aqueous dispersions concentration) need to be optimized [19, 102, 103]. Notably, to improve biocompatibility, pharmacokinetics, and biodegradability of these structures, studies ought to focus on environmentally benign synthesis approaches (with safer and non-hazardous agents), the hybridization of MXenes with biocompatible and biodegradable polymers (e.g., cellulose or chitosan), and the optimization of reaction/synthesis conditions. Surface functionalization of pristine MXenes with abundant functional groups on their surfaces deploying covalent and non-covalent modifications can also help to improve the targeting properties (selectivity/specificity), oxidation/thermal stability, and biocompatibility of MXenes, thus avoiding off-target effects and undesired defects (e.g., aggregation or accumulation) [60, 81, 92, 104–107]. In this context, the control of surface terminations, surface modifications using small molecules, surface-initiated polymerization, and single heteroatom approaches are some of the introduced strategies for surface functionalization of MXenes [108].

For the large-scale production of MXenes and their composites, studies need to focus on optimization of synthesis/reaction conditions and their repeatability to avoid structural defects and produce MXene-based nanozymes with excellent environmental stability, robust enzymatic activities, recyclability, suitable catalytic performances, and high specificity/selectivity [109–111]. In addition, simplicity, eco-friendly sustainable features, and cost effectiveness are crucial issues in translating the laboratory synthesis to industrial scale. Despite a variety of introduced synthesis techniques such as solvothermal treatment, calcination procedures, electrostatic self-assembly, hydrothermal synthesis, mechanical/ultrasonic mixing, chemical vapor deposition, among others, efforts are still required to focus on yield of production, feasible analyses, stability of final products, biosafety of chemical agents, and the reproducibility of processes [112, 113]. Some techniques such as mechanical/ultrasonic mixing strategies have shown interesting simplicity for fabrication of MXenes [112]. Meanwhile, MXenes with fascinating properties have been synthesized using electrostatic self-assembly and hydrothermal/solvothermal techniques [114]. Several etching approaches such as acid-, electrochemical-, and molten salt etching have been introduced. In synthesis techniques based on wet etching process, different etchants such as hydrogen fluoride, lithium fluoride, zinc chloride, etc., have been applied for manufacturing MXenes and their derivatives. Delamination procedures by assisting techniques of ultrasonication, flash freezing, and mechanical milling can efficiently applied for fabricating single- and few-layered MXenes. However, low stability and oxidation tendency are crucial challenges for etching and delamination of MXenes. In this context, still the adjustment of concentration and duration of etching processes ought to be addressed; higher temperatures, poor crystallinity, purity requirements, and high energy consumption are critical challenging issues that need to be resolved for the large scale production of MXenes; based on the external strain and the number of layers in the crystals and thin films of MXenes, their properties can be inventively adjusted [115, 116]. Chemical vapor deposition techniques can be considered for production of MXenes with high quality and defect-free structures [47], but only after optimization processes since these techniques may suffer from low yield of synthesis and complex treatment procedures [117].

## Conclusions and Future Outlooks

The development of nanozymes with lower manufacturing cost, higher catalytic stability, and ease of modification than natural enzymes ought to be a high priority for scientific research. Among the introduced nanozymes, MXene-based nanozymes have garnered considerable attention in the field of bio- and nanomedicine (especially, medical diagnostics) due to their unique catalytic and physicochemical properties. However, limitations regarding the peroxidase-like activity and sensitivity/selectivity may restrict further practical applications of pristine MXenes. Thus, developing an efficient surface engineering strategy is highly necessitated to obtain MXene-based nanozymes with multifunctionality and excellent performance. In this context, sulfur and nitrogen co-doping strategies can be applied to promote the peroxidase-like and electrochemical activity of MXene nanosheets, thus providing further active sites and improving the electron transport efficiency. Since related catalytic mechanisms using MXene-based composites (especially regarding the way of promoting reactions and the role of active sites on their surfaces) are not comprehensively illustrated, future studies should be directed toward improving the experimental/computational analyses as well as pre-/clinical studies to identify the underlying catalytic/enzymatic mechanisms, improve the properties/multifunctionality, and discover the advanced MXene-based nanozymes with responsive drug delivery and cancer nanotheranostic applications. The integration of nanotechnology with artificial intelligence can significantly help to expand the applications of these nanosystems in personalized medicine and nanomedicine.

MXenes exhibited suitable manageable catalytic performances, which can be further exploited for developing MXene-based biosensors with significant sensitivity and functionality. Notably, these structures can be contemplated as promising candidates in designing nanozymes with area-dependent electrocatalytic activity. Other 2D nanomaterials such as MOFs, transition metal dichalcogenides, layered double hydroxides, and transition metal oxides with enzyme-like features should be further explored along with MXenes for a variety of biomedical purposes owing to their alluring physicochemical properties of large specific surface area, ease of modification/functionalization, tuneable composition, ultrathin thickness, etc. In addition, future studies ought to transition toward the design of novel MXene-based nanoplatforms with excellent dual enzyme-like (oxidase- and peroxidase-like) catalytic activities to mimic biofilm microenvironment. These materials with suitable photothermal conversion efficiency in NIR-II window and enhanced dual enzyme-like catalytic functions along with no noticeable off-target side effects can be applied for effective anti-infective nanotherapy.
